# Root hair plasticity in cereals under abiotic stress

**DOI:** 10.1111/nph.71404

**Published:** 2026-06-29

**Authors:** Yaping Zhou, Frank Hochholdinger

**Affiliations:** ^1^ INRES, Institute of Crop Science and Resource Conservation, Crop Functional Genomics University of Bonn Bonn 53117 Germany

**Keywords:** abiotic stress, cereals, developmental plasticity, genetics, root hair

## Abstract

Abiotic stress is a constant threat to crop growth and yield in the context of climate change. Root systems that can best adapt their architecture to environmental challenges contribute to enhanced sustainability and resilience in crop production. As the outermost part of the root system with direct contact to the soil environment, root hairs are directly exposed to external stimuli and play a crucial role in responding to abiotic stresses by their developmental adaptation. Here, we review recently discovered genes regulating root hair plasticity under abiotic stress in cereals. Moreover, we discuss possible mechanisms by which abiotic stress‐derived signals can modulate cereal root hairs plasticity and enhance plant performance. Finally, we advocate using this knowledge to advance our understanding of the regulation of abiotic stress‐induced root hair plasticity in cereals, to optimize soil resource capture and to ultimately develop more stress‐adaptive crops, thus ensuring sustainable crop production under unfavorable conditions.

## Graphical abstract

An integrative perspective on how to study the regulation of root hair plasticity to increase the resilience of cereals to abiotic stress.

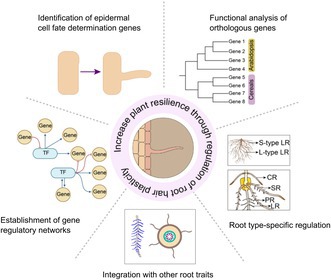

## 概括

在气候变化背景下，非生物胁迫持续威胁作物生长和产量。作物根系对环境变化的适应能力是维持作物生产力和增强农业系统韧性的重要基础。根毛作为根系最外层并直接与土壤环境接触的结构，直接暴露于外界刺激之下，并通过发育上的适应性变化在应答非生物胁迫过程中发挥关键作用。本文综述了近年来在禾本科作物中发现的调控非生物胁迫条件下根毛可塑性的关键基因，并探讨了非生物胁迫信号调控禾本科作物根毛可塑性及促进植物适应性的潜在机制。最后，我们倡导充分利用这些研究成果，进一步解析禾本科作物非生物胁迫诱导根毛可塑性的调控机制，优化土壤资源获取能力，并最终培育更具抗逆性的作物品种，从而保障不利环境条件下的农业可持续生产。

## Development and significance of root hairs in cereals

Climate change and unpredictable weather conditions substantially threaten global agriculture (Verma *et al*., 2024). Enhancing agricultural resilience requires more robust crops that can efficiently use soil resources and are better adapted to environmental changes (Foley *et al*., 2011). Under these challenging conditions, plant roots play a pivotal role in foraging soil for nutrients and water.

**Fig. 1:**
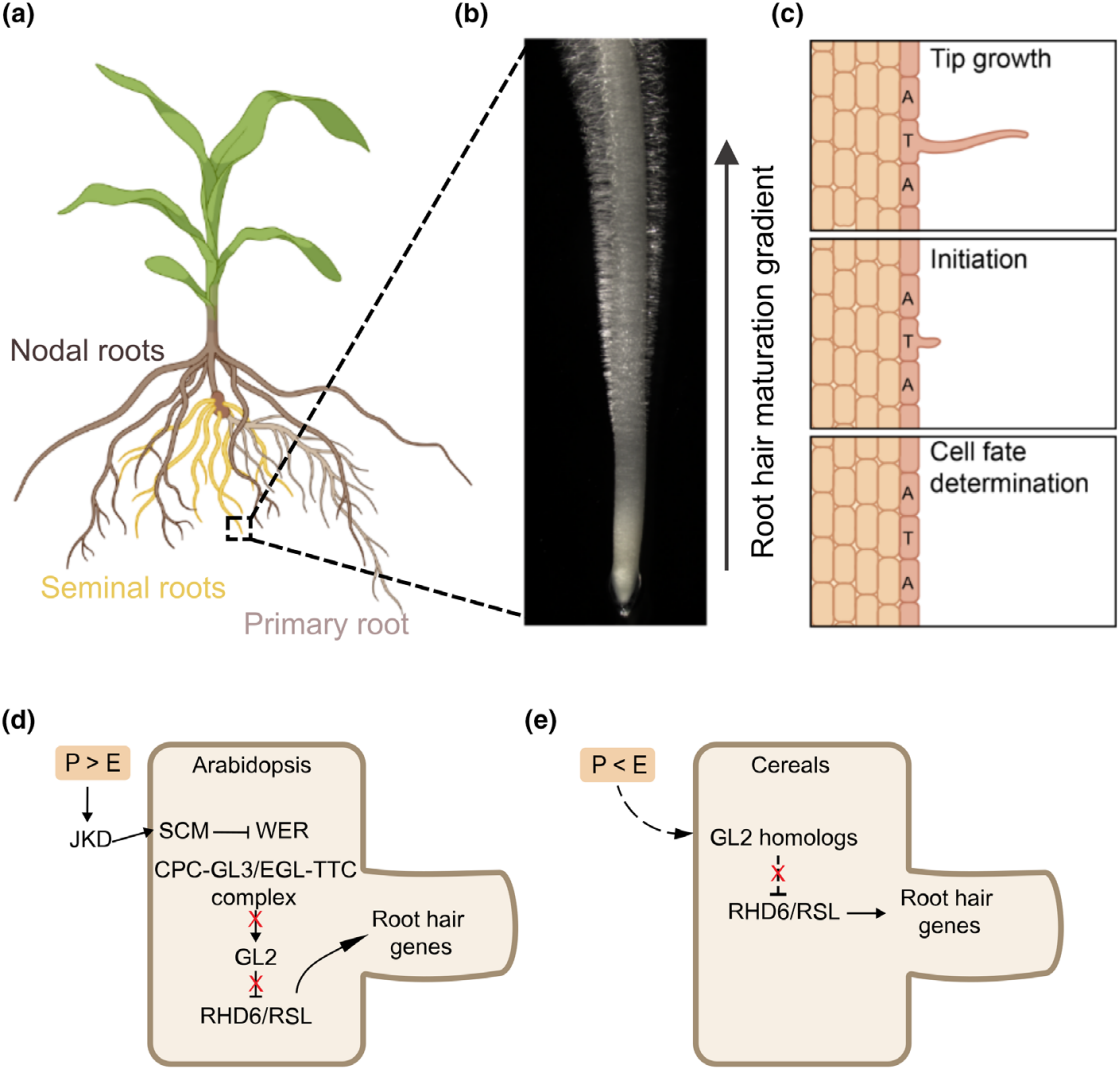
Root system architecture and root hair development in cereals. (a) Cereals form different root types, including the primary root, seminal roots, nodal roots and their lateral roots, which emerge from all main root types. (b) Root hairs (RH) are present on all root types and gradually elongate from the rootward to the shootward direction. (c) Epidermal cell types and three main stages of root hair development. The root epidermal cells consist of two cell types: tricoblasts (T), which differentiate into root hairs, and atricoblasts (A), which do not develop root hairs. Root hair development can be divided into three main stages: root hair cell fate determination, root hair initiation and root hair tip growth. (d) Regulation of root hair developmental patterning in Arabidopsis. (e) Regulation of root hair developmental patterning in cereals. Arrows indicate transcriptional activation; blunted lines denote transcriptional repression. Dashed arrows and dashed blunted lines represent putative regulatory interactions. Red crosses indicate blockage of the respective pathways. CPC‐GL3/EGL3‐TTG, CAPRICE–GLABRA3/ENHANCER OF GLABRA3‐TRANSPARENT TESTA GLABRA; E, environmental signals; JKD, JACKDAW; P, positional signals; RHD6, Root Hair Defective 6; RSL, RHD6‐LIKE; SCM, SCRAMBLED; WER, WEREWOLF. The maize plant icon in this figure was created in https://BioRender.com (https://BioRender.com/iodcbm5).

Root system architecture (RSA) is the three‐dimensional spatial configuration of different root types formed during development under the influence of external cues from the soil environment (Dresselhaus *et al*., 2025). Cereal crops develop a fibrous root system composed of multiple root types, including embryonic primary roots, seminal roots and post embryonic nodal roots and their lateral roots (Fig. 1a; Hochholdinger *et al*., 2018). The formation of root hairs on any of these root types significantly increases the root surface area for soil resource capturing (Fig. 1b; Dolan, 2017; Tsang *et al*., 2024). Moreover, root hair development is a complex process and mainly includes three stages: root hair cell fate determination, root hair initiation and root hair tip growth (Fig. 1c; Bibikova & Gilroy, 2003). All these processes are controlled by both genetic regulators and environmental factors. Ultimately, dynamic modifications of root hair morphogenesis under unfavorable conditions enhance root hair functions and improve plant performance (Müller & Schmidt, 2004).

Root hairs play a crucial role in helping plants to adapt to abiotic stress and to establish interactions with soil microorganisms (Datta *et al*., 2011; Vissenberg *et al*., 2020). Root hairs can enlarge the root surface area and create a favorable niche for colonization by beneficial microorganisms, such as plant growth‐promoting rhizobacteria (PGPR) and arbuscular mycorrhiza fungi (AMF). For instance, root hair traits are positively correlated with AMF colonization, which enhances plant performance under drought (Zhang *et al*., 2019). Moreover, one mechanism by which root hair‐associated microorganisms contribute to stress adaptation involves the release of nutrient‐mobilizing metabolites (Munir *et al*., 2022). These metabolites enhance the direct acquisition of nutrients. Furthermore, the synthesis of auxin by microbes constitutes an important mechanism through which bacteria influence root development (Finkel *et al*., 2020). Many associated microorganisms produce phytohormones, such as auxin, which stimulate root hair proliferation and enhance stress adaptation (Munir *et al*., 2022). In addition, plants also release root exudates to modulate rhizosphere bacterial communities in response to stress, with root hairs playing a pivotal role (Holz *et al*., 2018). A genetic study revealed that root hair regulators influence the assembly of the root microbiome under drought stress and contribute to microbiome‐mediated stress alleviation (Wang *et al*., 2024).

Root hairs are formed from specialized epidermal cells called trichoblasts. The pattern of root hair development varies between plant species (Osmont *et al*., 2007). In Arabidopsis, root hairs arise from epidermal cells located over the intercellular spaces between underlying cortical cells (Schiefelbein, 2000). This epidermal cell patterning and specification are orchestrated by positional cues through an integrated regulatory framework (Fig. 1d). First, the zinc finger protein JACKDAW (JKD) functions upstream of the receptor‐like kinase SCRAMBLED (SCM) to initiate position‐dependent signaling (Hassan *et al*., 2010). Subsequently, this signaling cascade represses the MYB transcription factor *WEREWOLF* (*WER*), resulting in the release of inhibition on the CAPRICE–GLABRA3/ENHANCER OF GLABRA3‐TRANSPARENT TESTA GLABRA (CPC‐GL3/EGL3‐TTG) protein complex (Salazar‐Henao *et al*., 2016). The formation and activation of this protein complex further reduce the expression of *GLABRA2* (*GL2*), a key negative regulator of root hair formation. Consequently, activation of the Root Hair Defective 6 (RHD6) and RHD6‐LIKE (RSL) transcriptional cascade in specified root hair cells drives the expression of genes required for root hair morphogenesis and ultimately initiates root hair outgrowth (Kohli *et al*., 2022).

In the cereal species, root hair pattering is largely position‐independent and does not follow the strict positional arrangement observed in Arabidopsis (Nestler *et al*., 2016; Zhang *et al*., 2023). Root hair and hairless cells alternate along epidermal cell files, resulting in roughly equal proportions (Dolan, 2017). In contrast to Arabidopsis, where epidermal cell fate is specified by well‐characterized positional cues mediated through a GL2‐centered genetic network, controlling root hair formation, the mechanisms governing epidermal patterning in cereals remain largely unknown. Moreover, whether a GL2‐like master regulator exists to determine epidermal cell fate in cereals is still an open question. Nevertheless, numerous studies indicate that the later stages of root hair differentiation are regulated by a conserved regulatory pathway involving RHD6 and RSL‐like transcription factors across species (Fig. 1e; Kohli *et al*., 2022; Tsang *et al*., 2024). Root hair development is controlled by the coordinated action of genetic pathways and environmental signals. Given both the conservation and divergence in epidermal cell fate specification mechanisms, it is likely that the relative contribution of environmental factors to root hair formation differs substantially between Arabidopsis and cereals.

Root hair growth, density and morphology are key components of RSA. Compared with optimal conditions, cereal root hairs contribute more substantially to water and mineral uptake when subjected to water or a nutrient constraint condition (Marzec *et al*., 2015; Kwasniewski *et al*., 2016; Klamer *et al*., 2019). Moreover, anatomical traits such as root hair length are increasingly recognized as important contributors to abiotic stress tolerance in crops (Cornelis & Hazak, 2021; Cai *et al*., 2022; Kohli *et al*., 2022). Furthermore, root hairs are proposed as a promising breeding target for sustainable crop production (Tsang *et al*., 2024). Only recently, the analysis of genetic regulators of root hair plasticity under nutrient deficiency, temperature stress, drought and salinity have been initiated in cereals. Here, we provide an update on the genetic components controlling root hair plasticity in response to these abiotic stress factors. Additionally, we provide novel research perspectives to maximize root hair functionality under abiotic stress for sustainable agriculture.

## Cereal root hair plasticity and abiotic stress responses

Root hair plasticity represents an essential component of a broader set of phenotypically plastic traits. It refers to the ability of root hairs to dynamically adjust their length, density and spatial distribution in response to environmental stimuli. The utilization of root hair plasticity promotes root growth and stress adaptation under adverse conditions (Zhu *et al*., 2010). Moreover, environmental signals induced by nutrient deficiency, drought, salinity and temperature stress significantly impact root hair performance by reprogramming gene regulatory networks, affecting hormonal and reactive oxygen species (ROS) levels (Shibata & Sugimoto, 2019; Vissenberg *et al*., 2020; Singh *et al*., 2024). This review aimed to highlight recent advances in the molecular and cellular mechanisms governing root hair plasticity, with a primary focus on changes in root hair length, density and developmental patterning under various abiotic stress conditions. These plastic responses are critical for plant adaptation and survival under fluctuating environments.

## Impact of drought stress on root hair plasticity in cereals

**Fig. 2:**
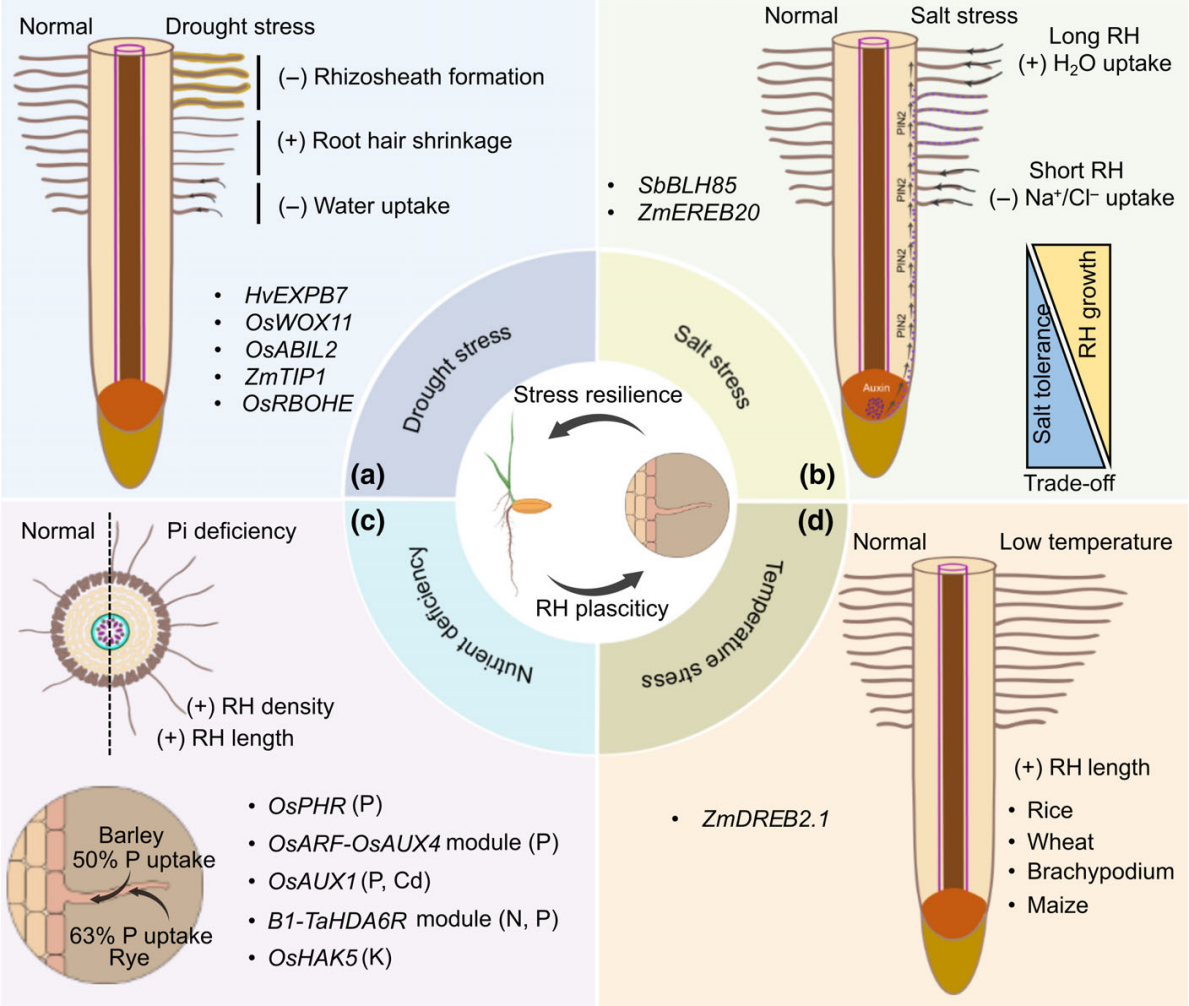
Root hair plasticity in response to various abiotic environmental stresses. (a) Drought stress induces root hair shrinkage and compromises their function in water uptake. Moreover, the formation of the rhizosheath contributes to plant resilience to drought stress. Root hair length and density were reduced under drought, thereby negatively influencing the formation of the rhizosheath. (b) Under salt stress, auxin originating from the root apex is transported to the mature root hair zone through the epidermis by the PIN2 transporter, thereby facilitating root hair elongation, thus enhancing water uptake. On the contrary, shorter root hairs limit Na^+^ and Cl^−^ uptake and accumulation in the root. Hence, the degree of root hair elongation under salt conditions reflects a trade‐off between water uptake and salt tolerance. (c) Phosphate (Pi) deficiency triggers root hair initiation and elongation, and greatly enhances Pi uptake in barley and rye. (d) Low temperatures trigger root hair growth in rice, wheat and Brachypodium, whereas root hair growth in maize is dynamically regulated by cold stress and displays genotype variation. The cereal genes previously identified to regulate root hair developmental plasticity under each abiotic stress are listed in the corresponding boxes. +, positive effect; −, negative effect. The rice plant icon in this figure was created in https://BioRender.com (https://BioRender.com/flwc5g9).

Drought induces a gradual disconnection of roots from soil with root hair shrinkage as the first step of root response to progressive soil drying (Duddek *et al*., 2022). Root hair shrinkage determines the root‐soil contact in the rhizosphere and water uptake (Fig. 2a; Duddek *et al*., 2023). Nevertheless, under drought stress conditions, root hair growth and development exhibit considerable plasticity, resulting in changes in root hair morphology and function that contribute to drought tolerance in crops (Karlova *et al*., 2021). Increasing root hair length and density maintain higher hydraulic continuity between roots and soil and improve water acquisition in drying soil (Shoaib *et al*., 2022). For instance, drought treatment significantly increases root hair length and density in maize (Li *et al*., 2017). Moreover, wheat drought tolerant genotypes with increased root hair length and density under polyethylene glycol (PEG) simulated drought conditions contribute to improved drought tolerance (Robin *et al*., 2021). Furthermore, interactions between long, dense and shrinkage‐resistant root hairs enhance drought resilience by maintaining root‐soil hydraulic connectivity, improving effective water absorption and delaying stomatal closure during soil drying (Cai *et al*., 2026). Collectively, drought‐induced adaptive changes in root hair morphology facilitate root‐soil interaction and resource absorption under water‐deficit condition, which also underscores the functional significance of root hair plasticity under drought.

Root hair plasticity can further shape rhizosheath development through dynamic modulation of root hair morphology and function. These modifications contribute to improved water retention and nutrient acquisition under drought conditions. Root hairs are widely recognized as key determinants for rhizosheath formation, a soil layer that adheres tightly to the root surface (Brown *et al*., 2017; He *et al*., 2025). The formation of the rhizosheath mitigates the development of air gaps around roots under harsh, dry environments, thereby increasing plant tolerance to drought stress and nutrient deficiency (Pang *et al*., 2017). Root hair length greatly influences specific rhizosheath formation and improves plant resilience to drought stress (Fig. 2a; Pang *et al*., 2017; Zhang *et al*., 2020). While the formation of the rhizosheath is influenced by multiple factors, including root hairs, soil physical properties, root exudation and microbial activity, the presence of root hairs is a prerequisite (Li *et al*., 2024). When root hairs are present, their development has a greater impact on rhizosheath formation than root exudate adhesiveness (Burak *et al*., 2021). Moreover, among the 58 examined species, no rhizosheath was observed on those species lacking root hairs (Brown *et al*., 2017). Furthermore, several studies demonstrated that auxin mediates root hair–microbe interactions to increase rhizosheath formation under soil drying. For instance, an auxin‐producing *Pseudomonas* strain induced root hair elongation and played a critical role in rice rhizosheath formation and drought tolerance (Xu *et al*., 2025). Barley enriched in the rhizosheath with the bacterial species *C. culicis* and *P. polymyxa* produce auxin (IAA) for root hair growth and thereby enhance rhizosheath formation for water use (Xu *et al*., 2023). In addition, abscisic acid (ABA) promotes rhizosheath formation under moderately dry soil by stimulating root hair elongation to offset drought stress in barley (Zhang *et al*., 2021).

Root hairs are not only essential for soil resource exploration and facilitating root‐microbe interactions but also serve as messengers for environmental signal sensing. Plasma membrane localized receptors or sensors perceive stress signals and subsequently transmit them to the cells through ROS or Ca^2+^ signals, further regulating downstream gene expression (Ravi *et al*., 2023). These signaling pathways are considered conserved regulatory mechanisms involved in stress sensing and transduction. In barley, the expression of Rho‐like GTPases (ROPs), calcium signaling and ROS‐related genes is substantially altered in the root‐hairless mutant *rhi1.a*, suggesting a potential role of root hairs in drought stress sensing (Kwasniewski *et al*., 2016).

In addition, a number of functional regulators involved in drought‐induced root hair plasticity have been identified in cereal crops (Fig. 2a). Importantly, these regulators mediate adaptive modulation of root hair traits in response to drought stress, while some also participate in basal root hair development. For instance, in Tibetan wild barley, *HvEXPB7* encodes a *β*‐expansin protein that is significantly induced under drought stress. This gene promotes drought‐induced root hair elongation and improves drought tolerance (He *et al*., 2015). In rice, *ABSCISIC ACID‐INSENSITIVE LIKE 2* (*OsABIL2*) negatively regulates root architecture via ABA signaling. Overexpression of *OsABIL2* impairs ABA‐induced root hair development and drought resistance, suggesting a role for ABA‐mediated root hair plasticity in drought responses (C. Li *et al*., 2015). Although the *RESPIRATORY BURST OXIDASE HOMOLOGUE* (*OsRBOHE*) functions in root hair formation independently of stress, it facilitates drought resistance through ROS‐mediated control of root hair development in rice (Zhao *et al*., 2024). In addition, the drought‐responsive gene *WUSCHEL*‐related homeobox gene *WOX11* has been shown to regulate crown root development and cytokinin signaling homeostasis in crown roots (Zhao *et al*., 2015), while also promoting root hair growth and enhancing drought resistance in rice (Cheng *et al*., 2016). In maize, natural variation of *TIP GROWTH DEFECTIVE 1/PAT24* (*ZmTIP1*) is significantly associated with drought tolerance (Wang *et al*., 2016). *ZmTIP1* encodes a functional S‐acyltransferase that mediates the S‐acylation modifications of the calcium‐dependent protein kinase (ZmCPK9) and positively regulates root hair growth. These findings demonstrate that *ZmTIP1* enhances drought tolerance by promoting root hair growth and efficient water uptake (Zhang *et al*., 2020).

## Impact of salt stress on root hair plasticity in cereals

Root hair proliferation under salt stress represents a trade‐off between adaptive benefits and potential costs. High salinity inhibits root hair growth by reducing their length and density in wheat (Robin *et al*., 2016) and Arabidopsis (Liu *et al*., 2023). This temporary inhibition of root hair growth reduces excessive uptake of toxic ions and enables plants to adapt to salt stress (Fig. 2b). On the contrary, maintaining growth of root hairs under salt stress conditions confers salt tolerance by extending the root surface area for water and nutrient uptake (Fig. 2b; Robin *et al*., 2016). The overall effect of root hair development on salt tolerance is context‐dependent and largely determined by the capacity of the plant to maintain ionic homeostasis, particularly the balance between Na^+^ uptake, exclusion and compartmentalization. In addition, the intensity and duration of salt stress (Wang *et al*., 2008), species‐specific differences (Verma *et al*., 2026) and root types (Arif *et al*., 2019) substantially influence the root hair modification in response to salt stress. Hence, root hair plasticity under varying salt conditions is considered an important agronomic trait for breeding salt‐tolerant crops.

In Arabidopsis, salt stress affects the localization of the auxin transporter PIN‐FORMED2 (PIN2) and induces its endocytosis. This effect regulates the levels of auxin transport in the epidermis close to the initial site of root hair development and controls the root hair growth (Fig. 2b; Wang *et al*., 2019; Ibeas *et al*., 2024). Given the central role of auxin in regulating cereal growth and development in response to abiotic stress, similar auxin‐mediated regulatory mechanisms may also contribute to root hair plasticity in cereal crops under salt stress (Voytenko & Kosakivska, 2025). Several transcription factors are involved in salt stress response and root hair initiation and elongation by regulating auxin signaling in cereals (Fig. 2b). For instance, sorghum *BASIC HELIX–LOOP–HELIX 85* (S*bbHLH85*) negatively regulates salt tolerance while promoting root hair elongation and density via ABA‐ and auxin signaling pathways (Song *et al*., 2022). Increased root hair growth was associated with enhanced Na^+^ uptake and salt sensitivity, suggesting a potential role of stress‐induced root hair plasticity in modulating ion uptake under saline conditions. SbbHLH85 also interacts with the phosphate (Pi) transporter chaperone PHF1 and affects Pi homeostasis, further linking root hair development with Pi uptake and salt tolerance. Moreover, the *ETHYLENE‐RESPONSIVE ELEMENT BINDING* factors *ZmEREB20* are induced by salt stress and alleviate salt‐induced inhibition of root hair growth by elevating ROS scavenging and auxin transport (Fu *et al*., 2021).

## Impact of nutrient deficiency on root hair plasticity in cereals

Root hairs facilitate soil nutrient acquisition and contribute to crop production, particularly under nutrient‐deficient conditions (Rongsawat *et al*., 2021; Fig. 2c) and contribute to the field performance of barley and maize (Wen *et al*., 2005; Hochholdinger *et al*., 2008; Zhang *et al*., 2018). Increased root hair length and density extend the nutrient accessible area in the rhizosphere and are beneficial for root uptake of low diffusible nutrients. In maize, the form and availability of nitrogen influence root hair plasticity, with longer root hairs being associated with a > 200% increase in biomass and nitrogen content under both glasshouse and field conditions (Saengwilai *et al*., 2021). Root hairs are particularly efficient in the acquisition of nonmobile Pi. Root hair proliferation allows roots to exploit otherwise nonaccessible stocks of Pi (Fig. 2c; Haling *et al*., 2014). In rice and maize, Pi starvation promotes root hair proliferation (Fig. 2c; Zhu *et al*., 2005; Giri *et al*., 2018). Several studies provide direct evidence that root hairs enhance the effectiveness of exploiting rhizosphere soil Pi. First, root hairs contribute up to 63% of the total Pi uptake from soil in rye (Fig. 2c; Gahoonia & Nielsen, 1998). Moreover, Pi uptake efficiency of a nonroot hair mutant decreased to 50% of their wild‐type (WT) plants in barley (Fig. 2c; Gahoonia *et al*., 2001). Furthermore, root hair length is highly correlated to the grain yield in barley under phosphorus deficient conditions (Gahoonia & Nielsen, 1998). Finally, previous studies combining synchrotron‐based X‐ray tomography with modelling of Pi diffusion demonstrate that contributions of root hairs and the main root surface to Pi uptake are comparable in wheat (Keyes *et al*., 2013).

In cereals, several genes have been identified that are essential for lowly mobile nutrients uptake via modifications of physical characteristics of root hairs (Fig. 2c). In rice, auxin signaling extensively regulates root hair plasticity in response to Pi deficiency. For example, rice *PHOSPHATE RESPONSE* (*OsPHR*) genes are functionally redundant and positively determine root hair formation by regulating auxin responsive genes (Guo *et al*., 2015). Moreover, *AUXIN RESPONSIVE FACTOR 12* (*OsARF12*) and the auxin influx carrier *AUX1/LAX4* (*OsAUX4*) negatively regulate root hair elongation (Ye *et al*., 2021), while OsAUX1 and the auxin efflux transporter OsPIN2 positively regulate root hair growth by redistributing auxin to root epidermal cells (Giri *et al*., 2018; Sun *et al*., 2019). In wheat, *B1* represses the expression of *TaRSL2* and *TaRSL4*, which are positive regulators of root hair elongation. A natural variant of *B1* promotes root hair length and nutrient uptake (N, P) by weakening B1‐TaHDA6‐mediated repression of *TaRSL2* and *TaRSL4* (Ke *et al*., 2025). Furthermore, genetic analyses have revealed that the activity of the potassium transporter OsHAK5 is highly associated with root hair length by modulating polar auxin transport (Yang *et al*., 2020). Finally, root hair‐specific expression of the auxin transporter OsAUX1 coordinates the expression of *OsCyCB1;1* and auxin distribution to regulate root hair development in response to cadmium stress (Yu *et al*., 2015).

Proteome analyses and genome‐wide association studies further contributed to the understanding of root hair plasticity regulation in response to abiotic stress. A proteomic analysis systematically examined protein dynamics in young maize root hairs in response to macro‐ and micronutrient deprivation (Z. Li *et al*., 2015). Moreover, a genome‐wide association study in bread wheat identified six candidate genes linked to Pi‐responsive root hair traits, including elongation and density (Maqbool *et al*., 2023). Among these, *TraesCS1A02G313600*, encoding a C_2_H_2_‐type domain containing protein, is associated with root hair elongation under low phosphorus availability. By contrast, *TraesCS3B02G132300*, which encodes a Glutaminyl‐tRNA synthetase, is linked to root hair density under high phosphorus conditions. These analyses provide valuable genetic resources to further elucidate the role of root hair plasticity in crop performance and to harness this trait for resilient agriculture.

## Impact of temperature stress on root hair plasticity of cereals

Soil temperature exhibits smaller fluctuations and higher minimum winter temperatures than air temperature (Fernández‐Pascual *et al*., 2015). In addition, increasing soil depth contributes to more stable soil temperatures (Fonseca de Lima *et al*., 2021). Thus, the root system is partially thermally buffered by its surrounding soil. However, ambient temperature fluctuation can impose heat or cold stress on root systems growing in the top soil layer, restricting their growth, soil exploration and function. As an important component of the root system, root hair growth and the plasticity of root hair patterning are dynamically controlled by low‐temperature changes (Pacheco *et al*., 2023).

Cold stress influences multiple aspects of root performance in cereals (Zhou *et al*., 2021). Low‐temperature triggers root hair growth in the monocots Brachypodium, rice and wheat (Fig. 2d; Shen *et al*., 2025; Urzua Lehuede *et al*., 2025). Moreover, cold tolerant rice genotypes have a higher root hair density than cold susceptible genotypes when exposed to cold treatment (Rativa *et al*., 2020). Furthermore, root hair growth in maize exhibits dynamic responses to cold stress (Zhou *et al*., 2024; Sommer *et al*., 2025). Exposure to mild low temperature initially suppresses root hair elongation, while prolonged exposure to stronger cold stress partially restores root hair growth to control level, suggesting an adaptive plastic response to low‐temperature stress (Fig. 2d; Zhou *et al*., 2024). Tissue‐ and cell type‐specific transcriptomic analyses further revealed that the dehydration response element‐binding protein 2.1 (ZmDREB2.1) negatively regulates cold‐induced root hair plasticity by coordinating the expression of the root hair defective genes *rth3* and *rth6* (Fig. 2d; Zhou *et al*., 2024). Genetic studies have identified several root hair defective genes (*rth3*, *rth5* and *rth6*) that function in a coordinated manner to regulate root hair development (Hochholdinger *et al*., 2018). Among them, *rth3* encodes a COBRA‐like protein associated with cell expansion and cell wall biosynthesis (Hochholdinger *et al*., 2008), whereas *rth6* encodes a cellulose synthase‐like D protein and is required for the transition from bulge formation to tip growth (Li *et al*., 2016). In addition, *rth5* contributes to apoplastic superoxide production and regulates the expression of cellulose biosynthesis genes upstream of *RTH3* and *RTH6* (Nestler *et al*., 2014).

## Knowledge gaps in research on abiotic stress‐induced root hair plasticity

**Fig. 3:**
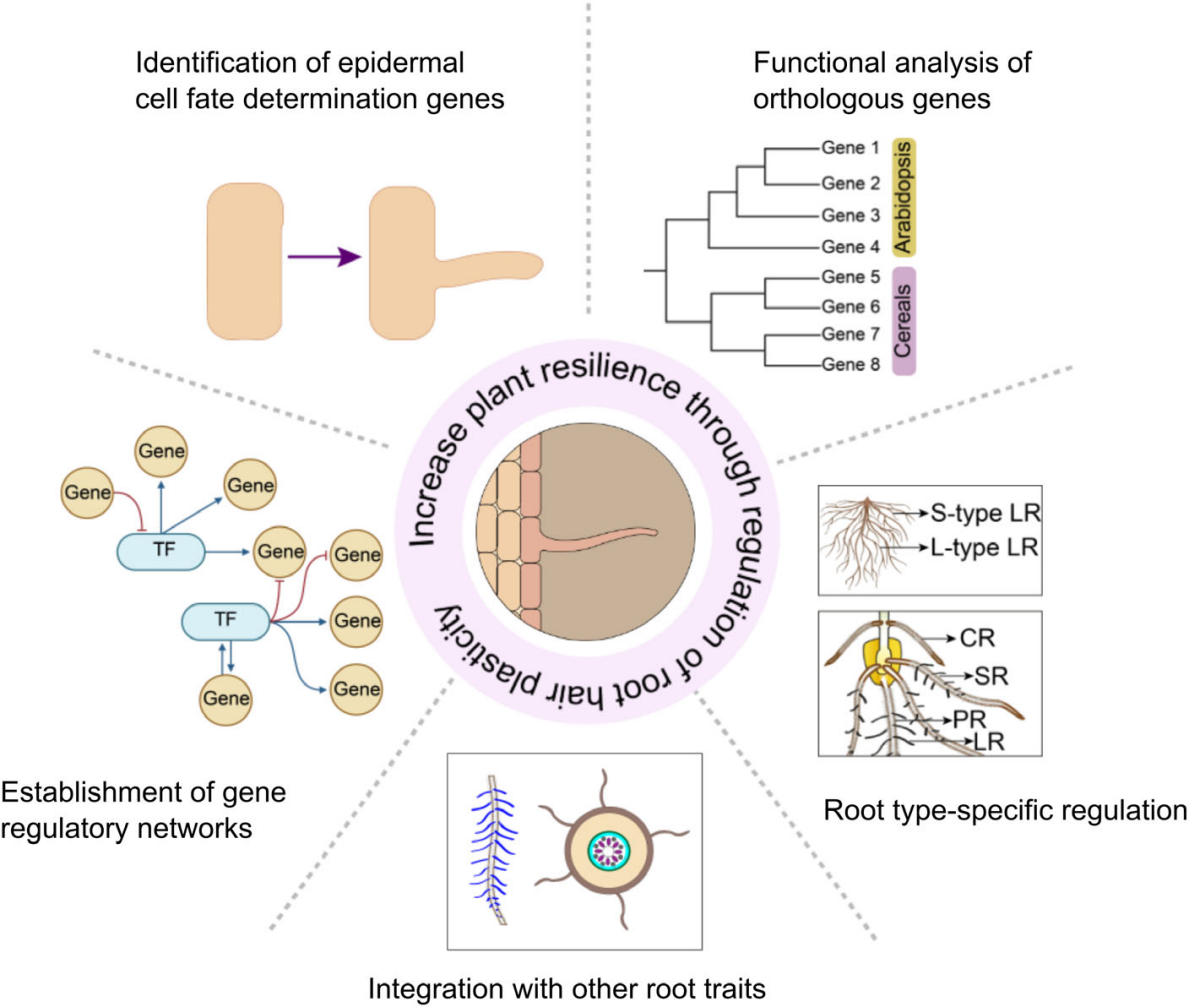
Integrative perspective on how to study the regulation of root hair plasticity to increase the resilience of cereals to abiotic stress.

The regulation of root hair plasticity to improve plant resilience under abiotic stress is still not fully understood, limiting the translation of this knowledge into agricultural practice (Fig. 3). First, root hair plasticity induced by abiotic stress is implicated in complex gene regulatory networks. Many studies on root hair plasticity in cereals primarily concentrated on nutrient deficiency and drought stress (Carminati *et al*., 2017; Lu *et al*., 2024). Nevertheless, our understanding of how abiotic stress shapes root hair plasticity in cereals still remains limited. Recently, genetic regulators controlling root hair formation and elongation for major cereal crops have been systematically compiled (Tsang *et al*., 2024). However, whether these genes are involved in hormonal regulation and interact with other genes under abiotic stress to facilitate crop production under stress conditions is not fully understood.

Moreover, cereal root systems comprise multiple root types. The coordination of different root types and various root traits maximizes overall root functionality. Thus far, abiotic stress‐induced root hair plasticity is mainly studied in young primary roots. For instance, root hair formation in rice differs between root types and is altered under artificial growth conditions (Nestler *et al*., 2016). Moreover, under drought stress conditions, maize root hair shrinkage is root type dependent, with turgid hairs observed on lateral roots near severely shrunk seminal roots (Duddek *et al*., 2022). This indicates that abiotic stresses could induce root type‐specific regulation of root hair plasticity. Thus, the precise mechanisms determining root hair plasticity in distinct root types under various stress conditions merit further investigation. In addition, root hair length and density in conjunction with other root traits, such as root branching and anatomy, represent key components of root functional attributes. Synergistic interactions among these traits improve root function and determine plant growth and productivity. Integrating these traits might lead to increased plant performance and stress resilience.

Furthermore, abiotic stress can disrupt the normal differentiation of root hairs. In Arabidopsis, the homeobox gene *GL2* mainly accumulates in nonroot hair cells, leading to the inhibition of root hair formation by repressing major root hair development‐related genes (*RHD6*, *RSL1*, *RSL2*, *Lj‐RHL1‐LIKE1* (*LRL1*) and *LRL2*; Lin *et al*., 2015). *GLABRA 2 EXPRESSION MODULATOR (GEM)* inhibits *GL2* activity by histone modification (Caro *et al*., 2007). Three homologs of *GEM* were identified in a root hair‐specific proteome analysis in maize, suggesting that they may be involved in the regulation of root hair development, possibly through mechanisms similar to those reported in Arabidopsis (Nestler *et al*., 2011). Genes involved in atrichoblast regulation in many other cereals are still largely unknown. In particular, it remains unclear whether the genes controlling root hair plasticity under abiotic stress conditions act through the GL2‐related pathway to reverse the root hair cell fate determination.

In addition, the function of many genes exhibits strong evolutionary conservation between diverse species. In comparison with Arabidopsis, the investigation of epidermal cell fate determination under abiotic stress conditions in cereals can be challenging due to the lack of epidermis marker lines. However, the emerging technologies of single cell sequencing and targeted and untargeted spatial transcriptomics enable the identification of key genes regulating epidermal cell fate determination in cereals. Moreover, root hair length and density exhibit large variations between species, accessions and environmental conditions (Rongsawat *et al*., 2021). Knowledge of the impact of abiotic stresses on root hair plasticity remains limited. Identification and functional validation of cereal orthologs of Arabidopsis root hair regulatory genes under abiotic stresses will enhance our understanding of root hair plasticity and its role in plant adaptation to challenging environments.

Finally, root hairs are increasingly considered as important interfaces for environmental stress sensing, and their growth may provide a proxy for the stress tolerance capacity of the root system. In maize, moderate cold stress significantly suppressed root hair elongation of the *Zmdreb2.1* overexpression lines, while enhancing the primary root cold tolerance, quantified as the ratio of the primary root growth rate under cold treatment to that under control conditions. Similarly, plants defective in root hair formation showed significantly enhanced primary root growth under cold conditions in maize and rice (Zhou *et al*., 2024). These findings indicate that reduced root hair length may provide an enhanced root cold tolerance; however, this effect likely depends on the functional roles of the underlying genes. These observations raise the possibility that root hairs may participate in early temperature sensing during cold stress responses. Similarly, compared with the WT, the barley root‐hairless mutant *rhl1.a* failed to activate water stress‐induced stress signaling and protective pathways, further supporting a potential role of root hairs as interfaces for drought sensing and stress signal transduction (Kwasniewski *et al*., 2016). However, despite these observations, the underlying mechanistic principles governing root hair‐mediated environmental sensing in cereals remain largely unresolved. In Arabidopsis, root hair sensing mechanisms are mediated by mechanical deformation of the plasma membrane in root hair cells, tip‐focused gradients and oscillations of ROS and Ca^2+^ (Novakovic *et al*., 2018; Zhang *et al*., 2022; Verma *et al*., 2026). Similar mechanisms may also operate in cereal crops, although direct experimental evidence remains limited. Moreover, RSA and its plasticity play a crucial role in plant survival and development under abiotic stress conditions (Karlova *et al*., 2021). RSA comprises multiple root traits and diverse cell types, of which root hairs represent only a small component of this complex system. In addition to root hairs, various root cell types possess the capacity to perceive and respond to stress signals. Furthermore, signaling pathways originating from different root traits and cell types are highly coordinated within the root, contributing to integrated environmental sensing at the whole root level. However, as the outermost cells of the root system, root hairs may respond rapidly to environmental changes, allowing for early perception of local cues. Future studies are needed to dissect how root hair‐mediated environmental sensing and responses are integrated with other root traits and cell types within the RSA to coordinate downstream stress responses.

## Concluding remarks

Root hair plasticity represents an important adaptive strategy influencing plant performance and resilience under various abiotic stress conditions. Recent studies identified novel regulators of root hair plasticity in cereals. Detailed knowledge of the regulation of abiotic stress‐induced root hair plasticity in cereals will help to develop stress‐adapted cereals.

## Competing interests

None declared.

## Author contributions

YZ and FH contributed equally to the conception, writing and revision of this review article.

## Disclaimer

The New Phytologist Foundation remains neutral with regard to jurisdictional claims in maps and in any institutional affiliations.
